# Supplementary material for: Identification of photoperiod-induced specific miRNAs in the adrenal glands of Sunite sheep (Ovis aries)
Source: Front Vet Sci. 2022 Jul 22;9:888207. doi: 10.3389/fvets.2022.888207 (PMC9354845; doi:10.3389/fvets.2022.888207)
Supplement: Supplementary file 1 [file Data_Sheet_1.ZIP › Supplementary file/miRDeep2 file.zip!/miRDeep2 file/Know_Structure/LP42A3/oar-mir-374a.pdf]

miRBase precursor : oar-mir-374a
Total read count : 21609
oar-miR-374a read count : 17655
remaining reads : 3954

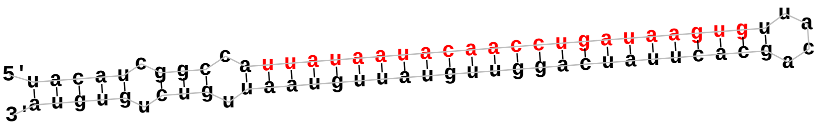

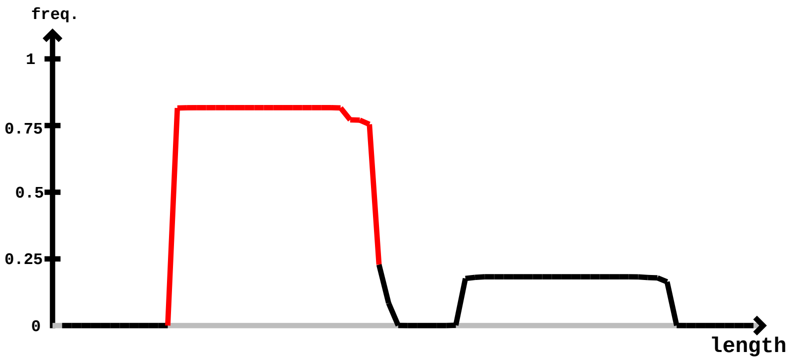


```
                  oar-miR-374a
5'- uacaucggccauuauaauacaaccugauaaguguuacagcacuuaucagguuguauuguaauugucugugua   -3'    exp
    (((((.(((.((((((((((((((((((((((((....)))))))))))))))))))))))).))).)))))    reads      mm         sample
    ..........auuauaauacaaccugauaagu........................................    1          0          seq
    ...........uuauaauacaaccugGu............................................    1          1          seq
    ...........uuauaauacaaccugau............................................    20         0          seq
    ...........uuauaauacaaccugGua...........................................    5          1          seq
    ...........uuauaauacaaccugauU...........................................    10         1          seq
    ...........uuaCaauacaaccugaua...........................................    12         1          seq
    ...........Cuauaauacaaccugaua...........................................    9          1          seq
    ...........uuauaauacaUccugaua...........................................    1          1          seq
    ...........uuaAaauacaaccugaua...........................................    10         1          seq
    ...........uuauUauacaaccugaua...........................................    7          1          seq
    ...........uCauaauacaaccugaua...........................................    3          1          seq
    ...........uuauaauacaaccugaua...........................................    908        0          seq
    ...........uuauaauacaaccugauaa..........................................    24         0          seq
    ...........uuauaauacaUccugauaa..........................................    1          1          seq
    ...........uuauaauacaaccugauaag.........................................    332        0          seq
    ...........uuauaauacaaccugGuaag.........................................    4          1          seq
    ...........uuaCaauacaaccugauaagu........................................    130        1          seq
    ...........uuauaauacaaccugauaagu........................................    11232      0          seq
    ...........uGauaauacaaccugauaagu........................................    1          1          seq
    ...........uuaCaauacaaccugauaagug.......................................    38         1          seq
    ...........uuauaauacaaccugauaagug.......................................    3076       0          seq
    ...........uuauaauacaaccugauaagugu......................................    1799       0          seq
    ...........uuauaauacaaccugauaaguguu.....................................    5          0          seq
    ............uauaauacaaccugauaagu........................................    8          0          seq
    ............uauaauacaaccugauaagug.......................................    2          0          seq
    ............uauaauacaaccugauaagugu......................................    10         0          seq
    ............uauaauacaaccugauaaguguu.....................................    1          0          seq
    .............auaauacaaccugauaagu........................................    1          0          seq
    .............auaauacaaccugauaagug.......................................    1          0          seq
    .............auaauacaaccugauaagugu......................................    2          0          seq
    .............auaauacaaccugauaaguguu.....................................    1          0          seq
    ...............aauacaaccugauaaguguuacagcacu.............................    1          0          seq
    ........................................acuuaucagguuguauugua............    3          0          seq
    ........................................acuuaucagguuguauuguaa...........    2          0          seq
    ........................................acuuaucagguuguauuguaau..........    10         0          seq
```

oar-miR-374a

```
uacaucggccauuauaauacaaccugauaaguguuacagcacuuaucagguuguauuguaauugucugugua
........................................acuuaucagguuguauuguaauu.........	9	0	seq
.........................................cuuCucagguuguauugu.............	1	1	seq
.........................................cuuaucagguuguauugu.............	8	0	seq
.........................................cuuaucagguuguauugua............	47	0	seq
.........................................cuuCucagguuguauugua............	1	1	seq
.........................................cuuaucagguuguauuAua............	1	1	seq
.........................................cuuaucagguuguauuguaa...........	26	0	seq
.........................................cuuaucagguuguauuguaau..........	305	0	seq
.........................................cuuaucagguuguauuguaauu.........	3400	0	seq
.........................................cuuaucagguuAuauuguaauu.........	1	1	seq
.........................................cuuaucagguuguauuguaauug........	2	0	seq
..........................................uuaucagguuguauuguaau..........	4	0	seq
..........................................uuaucagguuguauuguaauu.........	83	0	seq
..........................................uuaucagguuguauuguaauug........	1	0	seq
...........................................uaucagguuguauuguaau..........	1	0	seq
...........................................uaucagguuguauuguaauu.........	47	0	seq
...........................................uaucagguuguauuguaauug........	1	0	seq
```
